# Evaluating the Sensory Profile and Quality Traits of *Sparus aurata* Fed Sustainable Diets

**DOI:** 10.3390/foods15203590

**Published:** 2026-10-10

**Authors:** Marta Rodríguez-Soriano, Luis Noguera-Artiaga, Josep Álvar Calduch-Giner, Francisca Hernández, Ángel A. Carbonell-Barrachina, Ana M. Solivella-Poveda, Álvaro Belenguer, Jaume Pérez-Sánchez, Esther Sendra

**Affiliations:** 1 Instituto de Investigación e Innovación Agroalimentaria y Agroambiental (CIAGRO-UMH), Universidad Miguel Hernández, Carretera de Beniel, km 3.2, 03312 Orihuela, Alicante, Spain; 2 Fish Nutrigenomics and Integrative Biology, Institute of Aquaculture Torre de la Sal (IATS, CSIC), 12595 Ribera de Cabanes, Castellón, Spain

**Keywords:** alternative protein feeds, gilthead sea bream, insect meal, poultry meal, sustainable aquaculture, free amino acids, sensometrics

## Abstract

Sustainable Mediterranean aquaculture requires reducing fishmeal (FM) and fish oil (FO) use without compromising product quality. This study evaluated the effects of three isoenergetic and isoproteic diets—control fishmeal (FM), processed animal protein diet (PAP), and an alternative diet containing insect meal and microbial biomass (ALT)—on the physical, chemical and sensory properties of gilthead sea bream (Sparus aurata) at two catch weights (300 and 800 g). Texture and cooking weight loss were largely unaffected by diet, whereas PAP and ALT increased post-cooking yellowness and chroma. PAP showed the highest content of sweet and total free amino acids (FAAs), while umami FAAs remained unchanged. Gas chromatography identified 55 volatile organic compounds (VOCs), dominated by terpenes (49–54%, significantly higher in PAP and ALT), although lipid oxidation markers were similar. Sensory analysis revealed higher post-cooking chewiness and hardness in ALT, and enhanced umami for both alternative feeds. Consumer testing (*n* = 81) yielded high acceptance scores (>5/11-point scale) across treatments, with PAP scoring higher for oily flavor, textural oiliness, and aftertaste—an effect potentially linked to fatty acid composition rather than total fat content. Overall, replacing FM with PAP or ALT offers a sustainable feed strategy that preserves filet quality and consumer appeal.

## 1. Introduction

Driven by population growth and shifting dietary patterns, global demand for aquatic foods is projected to increase by 74% by 2050 [1]. As wild capture fisheries remain constrained by ecosystem productivity, aquaculture has emerged as a critical driver for global food security, achieving a record production of 130.9 million tons in 2022 [2,3]. Sustaining this expansion requires alignment with the United Nations Sustainable Development Goals (SDGs) and the FAO ‘Blue Transformation’ agenda, prioritizing resource efficiency, ecological resilience, and high food quality standards [3,4,5].

In Mediterranean aquaculture, gilthead sea bream (*Sparus aurata*) represents one of the most economically relevant species, reaching a global production of 311,600 t in 2023—a 2.9% increase compared to the previous year [6]. Key European markets, particularly Italy and Spain, represent the main destination hubs for sea bream consumption, reinforcing the economic necessity for sustainable farming strategies. Market demand for sea bream covers distinct size categories. Small fish (300 g) are sold for individual home meals, while larger specimens (800 g) serve the HORECA channel. Testing both sizes helps address real-world market segmentation challenges. However, sustaining this level of production relies heavily on the continuous optimization of dietary strategies. Among the factors that determine the quality of farmed fish, diet plays a central role because it directly influences tissue composition and many of the characteristics perceived by consumers in the final product. Although intrinsic and environmental factors, such as habitat, age, production system, and seasonality, exert a natural influence, dietary intake ultimately dictates final product quality [7,8,9].

Traditionally, aquafeeds have relied on fishmeal (FM) and fish oil (FO) due to their optimal nutritional profiles. However, the finite availability of these marine resources has driven continuous efforts to reduce their inclusion in aquafeeds. In gilthead sea bream, extensive research has demonstrated that both FM and FO can be substantially reduced using plant-derived ingredients. Satisfactory growth performance and nutrient utilization can be maintained through these reductions. This requires that the nutritional requirements for the species, particularly those related to essential amino acids and fatty acids, are adequately met [10,11,12,13]. Furthermore, some of the physiological constraints associated with high levels of marine ingredient replacement can be mitigated through the inclusion of functional feed additives [14,15]. Although these formulation strategies can modify tissue composition, such changes do not appear to compromise the nutritional value or overall quality of the final product [16].

More recently, research has moved towards the development of circular-economy-inspired formulations combining alternative protein and lipid sources, including processed animal protein (PAP), insect meals, microbial biomasses, microalga-derived products and aquaculture byproducts [17,18,19]. These novel formulations have demonstrated the ability to sustain growth performance and maintain fish health and welfare, while reducing the dependence on marine feed ingredients [20,21]. Moreover, evidence suggests that such dietary strategies can preserve filet quality traits and consumer acceptance, supporting their practical implementation in commercial aquaculture systems [22]. In line with this, recent studies in gilthead sea bream have evaluated three sustainable dietary formulations: a conventional fishmeal-based control diet (FM), a processed animal protein-based diet (PAP), and an alternative formulation (ALT) combining insect meal, microbial biomass and microalga-derived lipid sources. These dietary strategies have been shown to sustain growth performance, preserve liver and intestinal integrity, and maintain physiological robustness under farming conditions, even under unusually warm environmental scenarios, while eliciting distinct adaptive responses at metabolic, behavioral and transcriptional levels [23]. Furthermore, the same dietary formulations were shown to preserve the nutritional quality of the fillet by maintaining EPA + DHA levels and favorable human health lipid indices, while also ensuring a low transfer of contaminants to the edible product and maintaining food safety standards [24,25]. However, it remains critical to confirm whether these lipid-preserving feeds also maintain the physicochemical and sensory profile desired by consumers.

Consumer acceptance of farmed fish relies heavily on sensory perception, and alternative diets may modify organoleptic attributes [2]. Although the biological performance, physiological robustness and nutritional properties associated with these dietary formulations have already been established, their implications for consumer-perceived quality remain largely unexplored. Understanding whether dietary-induced changes are perceptible requires the combined use of analytical sensory methodologies and consumer-oriented approaches. Sensometrics provides the statistical framework needed to transform sensory data into actionable insights. Classical profiling by trained panels remains the gold standard for detecting subtle differences. However, rapid consumer techniques reported in the literature—such as Check-All-That-Apply (CATA) or Projective Mapping (PM)—may offer valuable complementary perspectives [2,4]. Integrating both enables a comprehensive evaluation of post-harvest quality attributes, including flavor, texture, aroma and appearance, which ultimately determine market acceptance of farmed fish [4]. In this context, the objective of the present study was to determine how sustainable feeding strategies influence the physical, chemical, and sensory quality of gilthead sea bream (*Sparus aurata*) by integrating instrumental measurements, flavor-related chemical traits, descriptive sensory analysis, and consumer perception.

## 2. Materials and Methods

### 2.1. Animal Ethics

All fish procedures were approved by the Ethics and Animal Welfare Committee of IATS, the CSIC Ethics Committee (permission 1295/2022), and Generalitat Valenciana (permission 2022-VSC-PEA-0230). They were carried out in the IATS’s registered aquaculture infrastructure facility (code ES120330001055) in accordance with the principles published in the European Animal Directive (2010/63/EU) and Spanish laws (Royal Decree RD53/2013) for the protection of animals used in scientific experiments.

### 2.2. Experimental Design

The trial was conducted as part of a comprehensive study evaluating sustainable feed formulations in gilthead sea bream. Details regarding rearing conditions, feeding trial and sampling strategy were previously described by [23,25], while the detailed formulation and ingredient composition of the experimental diets are provided in Table S1. Briefly, three isoenergetic and isoproteic diets were evaluated: (i) a fishmeal-based control diet (FM), (ii) a processed animal protein-based diet (PAP, incorporating poultry meal), and (iii) an alternative protein-based diet (ALT, incorporating insect meal and microbial biomass). Gilthead sea bream juveniles (initial body weight: 15 g) were reared from May 2022 to September 2023 in a continuous-flow system (3000 L tanks; two replicate tanks per diet) under natural temperature and photoperiod conditions at the IATS facilities (40°5′ N; 0°10′ E). Fish were fed near to visual satiety using automated feeders with pellet sizes adjusted to fish growth (2, 3, 4.5, and 6 mm). Fish were sampled in November 2022, with a body weight of 300 g, and at the end of the trial (September 2023), when fish reached a target market size of approximately 800 g. Ten fish per tank (*n* = 20 fish per dietary group) were randomly sampled at each sampling time, euthanized by concussion and subsequent decapitation, and processed. Right filets were dissected to evaluate meat quality parameters. A portion was homogenized, freeze-dried, and stored at −80 °C until free amino acid (FAA) analysis. Meanwhile, fresh right fillets were used immediately for physical (texture profile analysis, color, and percentage of cooking weight loss), volatile organic compound, and sensory analyses (descriptive and hedonic tests) (Figure S1).

### 2.3. Physical Analysis

Texture profile analysis (TPA) was performed using a texture analyzer (TA.XTplus, Stable Micro Systems, Surrey, UK) equipped with a compression plate probe (10 cm). Samples were prepared to uniform dimensions (2 × 2 cm) to ensure reproducibility and were equilibrated to room temperature (22 °C) prior to analysis. Each sample was subjected to a double compression test, simulating the action of mastication. The test parameters were set as follows: pre-test speed (1.0 mm/s), test speed (2.0 mm/s), and post-test speed (1.0 mm/s). Samples were compressed to 50% of their original height. The trigger force was set at a low threshold (3 g) to detect sample contact.

Color measurements were performed using a spectral colorimeter (Minolta CM-5, Osaka, Japan), and results were expressed in CIEL*a*b* coordinates. Fish samples were cooked in an oven at 180 °C for 4 min, until the core temperature reached 70 °C. Subsequently, samples were allowed to rest for 3 min prior to serving. Sample weight was recorded before and after cooking to determine cooking weight loss (expressed as %), using a balance with a precision of 0.001 g.

### 2.4. Free Amino Acids

Free amino acids (FAAs) were determined using a liquid chromatography–tandem mass spectrometry system (LC-MS/MS 8050, Shimadzu, Kyoto, Japan). Briefly, 0.2 g of freeze-dried sample was mixed with 400 µL of ethanol and homogenized for 10 min. The mixture was centrifuged at 12,000 rpm for 15 min at room temperature. The resulting supernatant was diluted (1:2, *v*/*v*) in ethanol, filtered through a 0.22 µm PTFE membrane, and collected in amber vials. Chromatographic separation was performed with an injection volume of 3 µL using a 30 min run. Identification and quantification were carried out using external standard calibration curves, and results were expressed as mg 100 g^−1^ of product [26]. Samples were analyzed using *n* = 4 biological replicates randomly selected per treatment group, with measurements performed in duplicate (analytical replicates). Detailed information regarding the analytical method and calibration standards is provided in Tables S2–S4.

To evaluate their potential contribution to the taste profile of the filets, individual FAAs were categorized based on their taste qualities [27]:

• Sweet FAAs: alanine, glycine, proline, serine, and threonine.
• Umami FAAs: aspartic acid and glutamic acid.
• Bitter FAAs: arginine, histidine, isoleucine, leucine, lysine, methionine, phenylalanine, tyrosine, and valine.

Total free amino acids (TFAAs) were calculated as the sum of all individual FAAs, which also included asparagine, cysteine, glutamine, 4-hydroxyproline, methionine sulfoxide, and tryptophan.

### 2.5. Volatile Organic Compounds

Volatile compound extraction was performed using an optimized protocol. Five grams of fish was transferred to 20 mL SPME vials with 4 mL of water (10% NaCl), and 20 µL of β-ionone (100 mg L^−1^) as internal standard. One cm length divinylbenzene/carboxen/polydimethylsiloxane (DVB/CAR/PDMS) fiber (50/30 μm) (Supelco, Bellefonte, PE, USA) was used for adsorption. Samples were incubated at 50 °C for 30 min under agitation (500 rpm) and fibers were exposed for another 20 min at the same temperature. Extraction was performed using a Shimadzu AOC-6000 Plus autosampler (Shimadzu Corporation, Kyoto, Japan) for precise temperature and agitation control. GC analysis was conducted using an InertCap Pure-WAX capillary column (30 m × 0.25 mm × 0.25 μm; Teknokroma, Barcelona, Spain). The oven program started at 40 °C, with an increase of 3 °C min^−1^ to 220 °C, and temperature was held for 10 min. Helium was used as the carrier gas at 1 mL min^−1^. Injection was performed in splitless mode at 250 °C. A Shimadzu TQ8040 NX mass spectrometer (Shimadzu Corporation, Kyoto, Japan) operated at temperatures of 200 °C for the ion source and 250 °C for the quadrupole, with 70 eV electron ionization. The scanning range was *m*/*z* 35–400 at 0.2 scans s^−1^. Volatile compounds were identified based on: (i) spectral matching with reference libraries (>90%) and (ii) calculating retention indices using a C8–C24 alkane series (Sigma-Aldrich, Steinheim, Germany) analyzed under the same chromatographic conditions as the samples. VOC relative abundance was calculated from chromatographic peak areas and expressed as the percentage contribution of each compound to the total integrated VOC peak area in each sample. Blanks were performed at the beginning and end of each sequence to monitor potential background signals, carryover, and contaminants originating from the SPME fiber or analytical system. Compounds detected in the blanks were excluded from the sample VOC profiles.

### 2.6. Descriptive Sensory Analysis

A trained panel of ten panelists (four males and six females) from the Miguel Hernández University (CIAGRO-UMH) performed the descriptive sensory analysis of fish samples. All panelists had great experience in descriptive sensory analysis of food products. Prior to the experimental evaluation, they completed five 1 h training sessions (5 h in total) specifically focused on gilthead sea bream. During these sessions, panelists were familiarized with the samples and sensory attributes and were calibrated through repeated sample evaluation and consensus. Duplicate samples were included in each training session to monitor within-panelist repeatability. Replicated samples were also included during the formal evaluation sessions to monitor repeatability throughout the study. Given the panelists’ previous experience and the consistency achieved during training, no further training sessions were considered necessary. The lexicon used was developed by the panel and included the following descriptors: (i) appearance: color, color uniformity, fat droplets, and lamellar structure; (ii) flavor: fish-ID, marine, fresh, fatty, earthy, vegetal, cooked, sweetness, sourness, bitterness, saltiness, umami, metallic, and rancid/ammonia; and (iii) texture: hardness, chewiness, adhesiveness, juiciness, oiliness, and aftertaste. The panelists used a 0–10-point scale for evaluation, where 0 represented extremely low intensity or absence and 10 represented extremely high intensity, with increments of 0.5. No external reference materials were used as anchor points for individual attributes. Instead, the interpretation of the attributes and the use of the intensity scale were standardized through consensus and repeated evaluation of representative gilthead sea bream samples during the training sessions. The analyses were conducted in a standardized tasting room with natural and white light, at a temperature of 22 ± 1 °C. Samples were served at 40 °C. Panelists were provided with water and breadsticks to cleanse their palates between samples.

### 2.7. Affective Sensory Analysis

Eighty-one regular fish consumers (at least once per week) participated in the affective study to evaluate the overall liking of the fish samples under investigation. The panel consisted of 41 women and 40 men; 51% were aged between 18 and 26 years, 28% between 27 and 42 years, and 20% between 43 and 60 years. A total of 14 sensory descriptors were evaluated: overall appearance, color, surface oiliness, fish/fresh/marine odor, oily odor, metallic odor, fish/fresh/marine flavor, oily flavor, metallic flavor, umami flavor, chewiness, juiciness, oiliness, and aftertaste. All descriptors were assessed using 11-point hedonic scales, where 0 corresponded to “dislike extremely” and 10 to “like extremely,” with 5 representing the midpoint (“neither like nor dislike”). The present study was approved by the ethical committee of Universidad Miguel Hernández de Elche (IAA.ESN.250203), and all trained panelists voluntarily consented to participate in the study. Informed consent was obtained from all subjects involved in the study.

### 2.8. Statistics

A one-way ANOVA and a Tukey test (*p* < 0.05) were performed to compare the means and determine whether there were significant differences between them. XLSTAT 2016 (Addinsoft, New York, NY, USA) was used to perform statistical analyses. Fish constituted the sampling units for physicochemical, chemical and sensory analyses. As fish originated from two replicate tanks per dietary treatment, potential tank-related effects cannot be completely excluded and should be considered when interpreting treatment differences. The assumptions of the ANOVA were verified prior to statistical analysis. Normality of residuals was assessed using the Shapiro–Wilk test, and homogeneity of variances was evaluated using Levene’s test. No significant deviations from these assumptions were detected (*p* > 0.05).

## 3. Results and Discussion

### 3.1. Physical Quality Parameters: Texture, Color, and Cooking Weight Loss

In the present study, the inclusion of alternative diets (PAP and ALT) did not impair the main mechanical properties of raw or cooked gilthead sea bream filets across both weight classes (300 and 800 g; Table 1 and Table 2, respectively).

In 300 g gilthead sea bream, no significant dietary differences were observed for any texture parameters, despite minor numerical increases in hardness and chewiness in alternative feeds. Similarly, in 800 g raw gilthead sea bream, overall texture remained statistically unaffected by diet, with post-cooking resilience being the only parameter showing a minor, albeit statistically significant, reduction in PAP and ALT compared to FM. The cohesiveness values were noticeably high due to the viscoelastic properties and rheological behavior of the fish filets during testing. The fillets underwent irreversible structural deformation following the first compression cycle, requiring greater work during the second compression. Because cohesiveness is calculated as the dimensionless ratio of the second to the first compression work area (Area 2/Area 1), this mechanical behavior yielded values exceeding 1.0. Consequently, chewiness and gumminess inherently reflected this same deviation, as direct mathematical derivatives of cohesiveness. The effects of dietary modifications on fish fillet texture are not always proportional to the magnitude of dietary reformulation, as substantial changes in ingredient composition may occur without translating into measurable differences in instrumental texture properties [16,28]. Accordingly, the lack of marked dietary effects on firmness, chewiness, and related texture parameters observed in the present study is consistent with previous findings in gilthead sea bream fed diets containing reduced levels of fishmeal and fish oil, as well as other alternative protein sources. Across these studies, dietary lipid and protein substitutions generally exert only minor effects on muscle physical properties, thereby preserving fillet texture and overall flesh quality [22,29]. Interestingly, this structural stability contrasts with dietary strategies involving specific functional fatty acids. For instance, a 0.8% inclusion of DHA in *Megalobrama amblycephala* significantly enhanced muscle hardness, springiness, shear force, and adhesiveness [27]. Overall, these findings indicate that dietary modifications generally have a limited influence on fillet texture, a pattern that is fully supported by the current results.

Instrumental color was not measured in 300 g gilthead sea bream as filet thickness was insufficient to ensure proper optical reading, leading to background interference. In raw 800 g gilthead sea bream, FM exhibited significantly higher yellowness (b*) and chroma (C*) than alternative feeds. However, thermal treatment markedly shifted this pattern: post-cooking, both PAP and ALT resulted in significantly higher b* and C* values than FM. Overall, lightness (L*) increased across all dietary treatments following thermal processing. Mechanistically, cooking denatures muscle proteins—particularly myosin—increasing L*, while heat-induced Maillard reactions and lipid interactions elevate b* [22]. Nevertheless, cooked ALT filets showed a slight reduction in L* compared to FM, likely associated with dietary insect and microbial pigments alongside subtle differences in surface moisture release during heating. Changes in baseline color, light reflectance, and carotenoid/lipid deposition are frequently documented when alternative proteins or oils replace marine feeds in sea bream [16,28]. In line with this, Álvarez et al. [16] reported a similar increase in C* for gilthead sea bream fed non-marine ingredients.

Regarding thermal processing, cooking weight loss showed no significant dietary differences, ranging from 5.09 to 5.38% and from 11.92 to 13.25% in gilthead sea bream weighing 300 and 800 g, respectively. This stability in water retention aligns with previous findings in gilthead sea bream, where substituting marine ingredients with vegetable oils [16] or poultry and insect meals [22] similarly preserved water-holding capacity and cooking yields.

### 3.2. Free Amino Acid Composition and Taste-Active Profiles

Free amino acid (FAA) contents were classified into taste-active categories (sweet, umami, and bitter) to correlate chemical profiles with sensory attributes (Table 3 for 300 g and Table 4 for 800 g gilthead sea bream).

Overall, the umami FAA category showed no significant differences among dietary treatments in either weight group (300 and 800 g). However, dietary formulations markedly influenced sweet FAA and total FAA (TFAA) contents across both size classes.

In 300 g gilthead sea bream (Table 3), the PAP diet induced the highest sweet FAA (24.38 mg 100 g^−1^) and TFAA (31.88 mg 100 g^−1^) contents, whereas ALT exhibited significantly lower values for both (16.99 and 23.07 mg 100 g^−1^) compared to PAP, remaining numerically below the FM control (21.62 and 28.15 mg 100 g^−1^, respectively). No significant differences were observed for bitter FAAs (2.26–2.77 mg 100 g^−1^). Regarding individual FAAs, glycine (Gly) and alanine (Ala) predominated across all diets, whereas arginine (Arg) and aspartic acid (Asp) were the least abundant. This lower muscle TFAA accumulation in ALT may reflect differences in amino acid turnover and partitioning rather than differences in growth performance, since the experimental diets were formulated to be isoenergetic and isoproteic and supported comparable productive performance under farming conditions [23]. This suggests that alterations in the muscle FAA pool do not necessarily reflect impaired nutritional status or reduced growth, but may instead result from diet-specific metabolic adjustments. Conversely, a 50% inclusion of insect meal was reported to increase muscle FAAs [30] and reduce growth due to lower protein efficiency [31].

In 800 g gilthead sea bream (Table 4), a similar trend was observed for PAP, which again displayed significantly higher sweet FAA (19.59 mg 100 g^−1^) and TFAA (28.22 mg 100 g^−1^) values than both FM (12.16 and 17.03 mg 100 g^−1^) and ALT (13.49 and 19.67 mg 100 g^−1^). Remarkably, TFAA content in PAP was approximately 66% higher than in FM at this weight. At 800 g fish, bitter FAAs were also significantly elevated in both PAP (1.83 mg 100 g^−1^) and ALT (1.65 mg 100 g^−1^) treatments compared to FM (0.97 mg 100 g^−1^), though no significant differences were detected between PAP and ALT. A similar pattern was observed at 800 g, with Gly and Ala remaining the highest and Arg and Asp the lowest. The overall depletion of TFAA levels observed as fish reached 800 g suggests that larger body size influences metabolic dynamics, promoting a more efficient protein utilization, with reductions of 39.5% in FM, 14.7% in ALT, and 11.5% in PAP compared to 300 g fish. While dietary poultry byproduct meal is known to maintain overall filet quality in gilthead sea bream [22,25,32], studies evaluating its impact on muscle FAA pools remain absent. To the best of our knowledge, this is the first work reporting muscle FAAs under dietary PAP inclusion, limiting direct comparisons. Although analyzing total amino acids was not the primary aim of this research, evaluating these profiles remains relevant for diet quality and protein biosynthesis [30]. Nevertheless, focusing on muscle FAAs serves as a direct indicator of protein utilization efficiency in the fillet. Beyond metabolism, muscle FAAs contribute significantly to fish flesh flavor, where these taste-active fractions (sweet, umami, and bitter) act as key precursors shaping fillet sensory perception [27,33].

### 3.3. Volatile Organic Compound Profile

A total of 55 volatile compounds were identified by GC analysis in 800 g gilthead sea bream, classified into nine chemical families: acids, alkanes, alcohols, aldehydes, alkenes, aromatic hydrocarbons, ketones, terpenes, and others (Table 5). Significant differences were observed across all chemical families, except for aldehydes (3.47–4.04%) and ketones (1.16–1.58%).

Generally, terpenes were the dominant chemical family in all treatments, accounting for approximately 49–54% of the total volatile compounds identified. Furthermore, the total terpene content was significantly higher in fish fed PAP and ALT diets than in those fed the FM diet (53.18 and 53.84 vs. 48.86, respectively). This finding suggests a strong dietary influence of alternative protein ingredients on the aroma profile of sea bream flesh. Terpenes are generally not synthesized in large amounts by fish and are mainly derived from dietary sources, accumulating in tissues after ingestion [34]. Therefore, the higher abundance observed in PAP and ALT groups may be related to the incorporation of plant-derived ingredients, vegetable oils, or metabolites present in the substrates used for insect production. Among the individual terpenes, D-limonene, γ-terpinene and β-pinene were particularly noteworthy. D-limonene was significantly higher in ALT-fed fish than in FM-fed fish (19.57 vs. 16.33), whereas β-pinene showed the opposite trend, reaching its highest concentration in FM fish. These compounds are typically associated with citrus, resinous and herbal aroma notes and often possess low odor thresholds, suggesting a potentially important contribution to the sensory differentiation among treatments. The elevated limonene level in ALT fish may indicate a greater transfer of terpene compounds originating indirectly from plant-based substrates used during insect rearing. As detailed in Table S1, the experimental feeds contained several plant-derived ingredients, including corn gluten meal, soybean meal, sunflower meal, wheat meal, whole peas, pea starch, and rapeseed oil, which represent potential dietary sources of plant-associated volatile compounds. D-limonene and other terpenes detected in muscle could therefore originate, at least partially, from these ingredients or, in insect-containing formulations, from compounds associated with the insect-rearing substrates.

In contrast, aldehydes and ketones, which are commonly regarded as markers of lipid oxidation, showed limited dietary effects. Total aldehyde levels did not differ significantly among treatments, and major oxidation-derived compounds such as hexanal, nonanal and decanal remained relatively stable across diets. This result is particularly relevant because aldehydes are frequently associated with rancid, fatty and oxidized aromas in fish products [35]. The absence of significant differences suggests that replacing fishmeal with plant, insect proteins or other animal proteins did not increase lipid oxidation in sea bream filets. This outcome is likely related to the similar lipid content and balanced formulation of experimental diets. Alcohols were more abundant overall in PAP and ALT fish than in FM fish (9.63 and 9.21 vs. 8.23, respectively). However, some alcohols associated with fatty acid oxidation, such as 1-hexanol, were significantly lower in PAP and ALT treatments. This indicates that the observed changes in the alcohol fraction cannot be attributed solely to oxidative processes but are more likely related to differences in metabolic pathways and precursor availability associated with the alternative protein ingredients [36]. The alternative diets also led to significant increases in several aliphatic and cyclic hydrocarbons. Total alkanes were significantly higher in PAP and ALT fish than in FM fish (7.74 and 7.35 vs. 5.14, respectively). Similarly, compounds such as pristane, tetradecane, cyclooctatetraene and cyclododecane were found at greater concentrations in fish receiving alternative protein sources. Although hydrocarbons generally possess high odor thresholds and therefore contribute less directly to aroma perception, they may serve as indicators of differences in dietary composition and metabolic processing of feed ingredients. An interesting finding was the carboxylic acids. At the individual compound level, several medium- and long-chain acids, including octanoic, nonanoic and decanoic acids, were present at higher concentrations in PAP and, to a lesser extent, ALT fish. Nevertheless, when considered as a chemical family, total acids were highest in FM treatment and progressively decreased in PAP and ALT fish. This apparent contradiction is largely explained by the markedly higher concentration of acetic acid in the FM group (4.43 vs. 2.17 and 1.56, respectively). Therefore, the reduction in total acid content observed in the alternative diets was mainly driven by lower acetic acid levels, despite increases in several other individual acids.

Taken together the above findings suggest that the differences observed are primarily related to the intrinsic properties of the feed ingredients rather than to changes in flesh oxidative stability. Previous studies have shown that dietary modifications in gilthead sea bream, specifically the replacement of fish oil with plant oils, alter the aromatic profile through changes in volatile compounds. However, these differences are not subsequently perceived by consumers, as the main sensory impact is determined by post-harvest storage conditions and product formulation [37,38,39]. These observations agree with the present results, where differences in VOC composition were not reflected in significant differences in freshness-related sensory descriptors.

### 3.4. Descriptive Sensory Analysis by Trained Panel

A descriptive sensory evaluation of cooked gilthead sea bream was performed by a trained panel to identify specific organoleptic modifications induced by the experimental diets across both commercial sizes (Table 6 and Table 7). Overall, the sensory evaluation revealed distinct size- and diet-dependent responses.

In 300 g gilthead sea bream (Table 6), visual differences were confined to fat droplets, where PAP (4.7) scored significantly lower than FM (8.1) and ALT (7.3). Although cooking weight loss was not statistically significant (Table 1), PAP showed the highest numerical thermal exudation (5.38%), which likely favored lipid loss into cooking juices rather than surface retention [40,41]. Regarding flavor, umami was the only descriptive attribute significantly affected (Table 6), with both alternative diets (PAP and ALT) scoring higher than FM. Interestingly, this contrasts with chemical data (Table 3), where umami FAAs showed no significant differences despite a higher numerical value in PAP. This perceptual enhancement likely reflects synergistic interactions between free amino acids, endogenous 5′-nucleotides (e.g., IMP), and thermally generated umami peptides upon cooking. Specifically, thermal processing triggers Maillard reactions that release umami peptide derivatives with exposed reactive hydroxyl or amino groups, amplifying taste receptor binding beyond baseline raw FAA levels [42]. Conversely, while sweet FAAs differed chemically (PAP > FM > ALT), descriptive sweetness showed no statistical differences (Table 6), though alternative diets remained numerically higher. This divergence may stem from taste masking. Higher umami levels can allosterically suppress sweet receptors (TAS1R2), reducing perceived sweetness [42]. Overall, while baseline FAA levels do not directly dictate perceived flavor intensity due to thermal matrix interactions, both alternative formulations generally enhanced taste profiles compared to FM. In terms of texture, ALT scored significantly higher than FM for chewiness (6.2 vs. 4.6) and aftertaste (7.9 vs. 6.8), with PAP displaying intermediate scores. The sensory texture results aligned with the raw instrumental TPA trends (Table 1), where ALT and PAP showed numerically higher baseline hardness and chewiness than FM. Thermal cooking amplified this structural firmness, making the higher chewiness of ALT statistically significant in the cooked filet (Table 6). In 800 g gilthead sea bream (Table 7), visual differences shifted to color, where PAP (3.6) scored significantly higher than FM (2.3) and ALT (2.1). This sensory observation aligns with the instrumental post-cooked color (Table 2), where both alternative feeds exhibited higher b* and C* than FM. No statistically significant variations were detected for any sensory flavor attribute. Interestingly, the higher terpene abundance observed analytically in the PAP and ALT treatments was not accompanied by increased herbal, vegetal, citrus, or related sensory notes. Thus, differences in the GC-MS volatile profile did not translate into perceptible differences in these sensory attributes. However, because volatiles were evaluated in terms of relative abundance rather than absolute concentration, odor activity values could not be calculated, and it cannot be established whether individual terpenes were above or below their perception thresholds in the cooked fish matrix. This distinction is relevant because odor thresholds are strongly dependent on the food matrix and may differ substantially from those obtained in water or other simplified systems. Nevertheless, PAP consistently displayed the highest numerical scores for sweetness, bitterness, and umami. This sensory pattern aligns well with the chemical FAA profiles (Table 4), where PAP exhibited significantly higher sweet, umami, and bitter fractions. For texture, significant differences were confined to hardness, with ALT (6.4) scoring significantly higher than FM (3.9), while PAP (5.5) showed intermediate values. This aligns with post-cooking instrumental TPA trends (Table 2), where ALT exhibited the highest cooked hardness (573 g). In summary, these results confirm that alternative formulations, particularly PAP, consistently enrich taste-active precursor pools over FM up to commercial size.

### 3.5. Consumer Acceptance and Hedonic Evaluation

**Figure 1:**
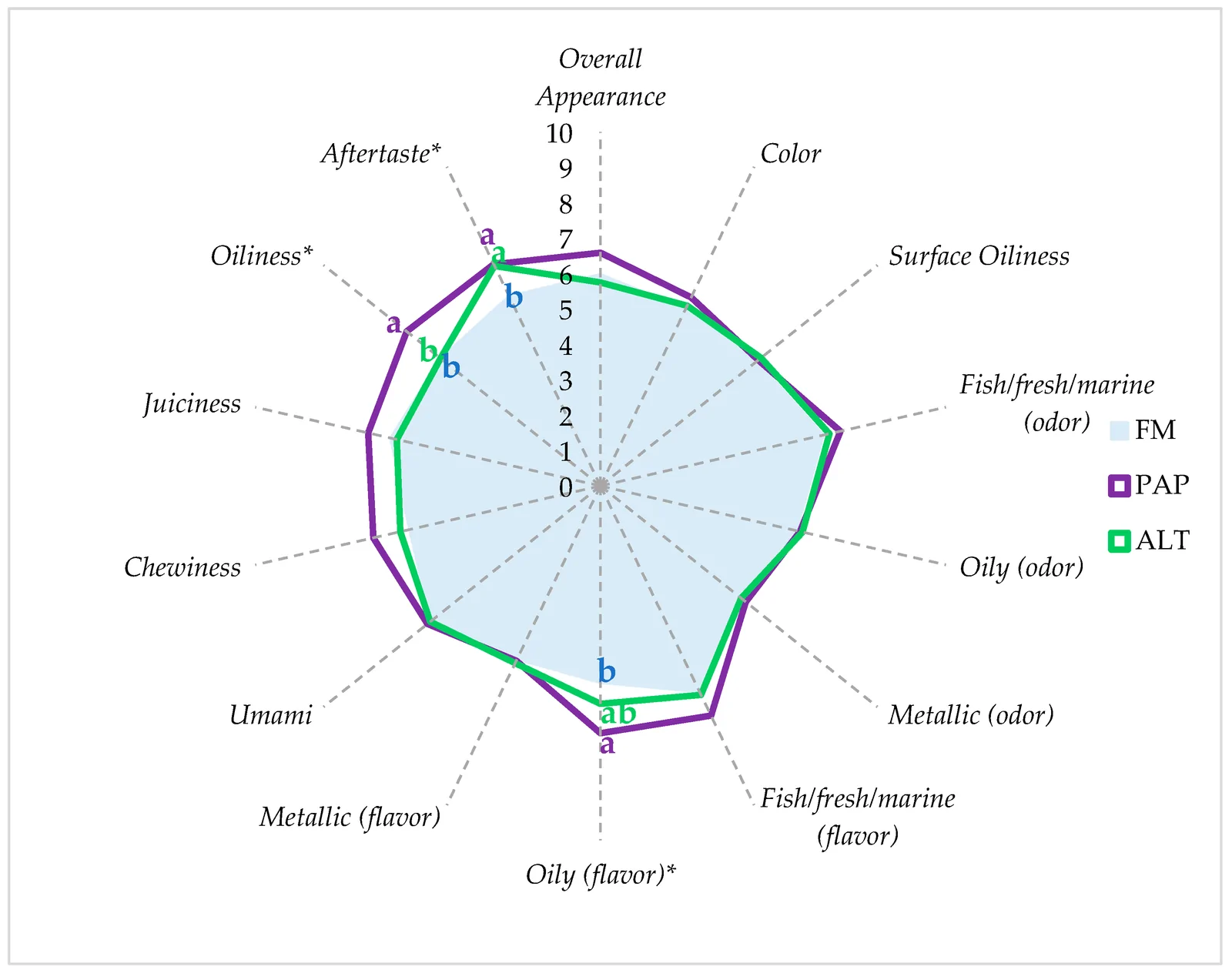
Consumer hedonic evaluation of cooked gilthead sea bream (800 g). Evaluation based on an 11-point hedonic scale (0 = “dislike extremely”, 5 = “neither like nor dislike”, 10 = “like extremely”). FM: Fish meal-based diet; PAP: processed animal proteins-based diet; ALT: microbial biomass and insect protein-based diet; *: *p* < 0.05. Different lowercase letters within the same row denote significant differences among dietary treatments according to Tukey’s HSD multiple range test (*p* < 0.05). Absence of letters indicates no significant differences.

Figure 1 illustrates the spider plot corresponding to the hedonic consumer evaluation of cooked gilthead sea bream (800 g), while the detailed numerical scores and statistical analysis are reported in Table S5.

Overall, all dietary treatments demonstrated high consumer acceptance, with all evaluated attributes exceeding the threshold of 5 points across the visual, odor, flavor, and texture categories. Regarding appearance and odor, no significant differences were detected among dietary groups for any attributes (*p* > 0.05). Within the flavor category, the only attribute displaying statistically significant variations was oily (*p* = 0.001), where the PAP group scored significantly higher (7.0) than FM (5.6), while ALT exhibited intermediate values (6.2). In terms of texture attributes, significant differences were confined to oiliness (*p* = 0.001) and aftertaste (*p* = 0.015). Specifically, PAP was perceived as markedly oilier (7.0) compared to both FM (5.8) and ALT (5.8). For aftertaste, PAP (7.0) also achieved the highest value, being significantly superior to FM (6.0) yet statistically comparable to ALT (6.9). This alignment between oily flavor and textural oiliness highlights a clear sensory consistency in the mouthfeel perception of the PAP diet. Interestingly, the enhanced perception of oily flavor and textural oiliness reported by consumers for the PAP diet was not driven by total muscle fat, as Sendra et al. [25] found no significant differences in crude fat among diets (7.66% for PAP, 8.67% for FM, and 8.51% for ALT). Instead, this heightened sensory oiliness aligns with the dietary-induced restructuring of the fatty acid profile—specifically, the significantly higher proportions of saturated fatty acids (24.4%), polyunsaturated fatty acids (PUFAs, 21.8%), and omega-3 fatty acids (17.2%) in PAP filets compared to FM (23.5%, 20.7%, and 15.6%, respectively) [25]. From an oral processing perspective, this fatty acid profile alters lipid melting dynamics at human oral temperature (37 °C), while amphiphilic salivary proteins promote in situ emulsification into oil-in-water droplets [43,44]. Modulated interfacial stability and colloidal interactions within the salivary film increase droplet adhesion to the oral mucosa and tongue epithelium, retarding lipid clearance and directly enhancing perceived textural oiliness, film viscosity, and lingering aftertaste during mastication [43,44].This suggests that the consumer perception of oiliness reflects the release and interaction of this distinct lipid matrix in the mouth during mastication, rather than the crude fat content in the muscle.

Taken together, these findings highlight the value of sensometrics in evaluating sustainable feed formulations. Identifying these specific organoleptic shifts is essential, as producers and manufacturers need to know how consumers perceive fish fed alternative feeds to remain competitive in the aquaculture market [4]. Ultimately, although the PAP diet showed the highest preference, both alternative formulations proved to be effective fishmeal replacements that preserve consumer appeal and support sustainable aquaculture.

## 4. Conclusions

This study demonstrates that FM and FO can be successfully substituted with processed animal proteins (PAP diet) or a combination of insect meal and microbial biomass (ALT diet) in gilthead sea bream diets without compromising muscle physical integrity or final consumer acceptance. Both alternative formulations preserved essential physical quality attributes, including mechanical texture profiles and water-holding capacity (cooking yield), while maintaining equivalent oxidative stability, as shown by stable volatile aldehyde profiles across treatments.

While thermal processing induced slight chromatic shifts toward higher yellowness (b*) and chroma (C*) in PAP and ALT filets, these visual alterations did not adversely affect organoleptic perception. Biochemically, the PAP diet enhanced muscle free amino acid pools—particularly sweet FAAs—and both alternative diets altered volatile terpene profiles, reflecting the direct metabolic integration of non-marine raw materials.

Sensory evaluations by both descriptive panels and regular consumers confirmed that PAP and ALT sustain high product quality. Consumer hedonic testing established that all diets exceeded acceptable liking thresholds, with PAP scoring highest in oily flavor and mouthfeel due to fatty acid restructuring rather than increased muscle lipid deposition. Consequently, PAP and ALT represent viable, scalable feed options that support the FAO ‘Blue Transformation’ agenda by reducing marine resource dependency while safeguarding the flavor, appearance, and market value of farmed gilthead sea bream. PAP-based diets benefit from the established availability of poultry-derived ingredients, whereas formulations incorporating insect meal and microbial biomass broaden the range of sustainable feed resources available to the aquaculture sector. Their widespread adoption will depend on market conditions and ingredient availability. The ability of both strategies to maintain product quality and consumer acceptance further supports their commercial implementation.

## Figures and Tables

**Table 1:** Raw texture parameters and weight loss percentage of gilthead sea bream (300 g) fed experimental diets.

Parameter	ANOVA (*p*-Value)	FM	PAP	ALT
Hardness (g)	0.351	1397 ± 394	1541 ± 289	1549 ± 335
Adhesiveness (g s)	0.631	−12.87 ± 3.64	−13.72 ± 3.64	−13.91 ± 3.00
Springiness	0.272	0.88 ± 0.01	0.88 ± 0.01	0.88 ± 0.01
Cohesiveness	0.923	2.27 ± 0.26	2.30 ± 0.28	2.26 ± 0.29
Gumminess	0.325	3157 ± 928	3526 ± 668	3464 ± 665
Chewiness	0.294	2769 ± 816	3112 ± 591	3051 ± 597
Resilience	0.455	0.30 ± 0.03	0.32 ± 0.04	0.31 ± 0.04
Cooking weight loss (%)	0.808	5.09 ± 1.12	5.38 ± 1.09	5.35 ± 1.21

FM: fishmeal-based diet; PAP: processed animal protein-based diet; ALT: microbial biomass and insect protein-based diet. Data are presented as means ± SD (*n* = 20). The ANOVA column indicates the *p*-value derived from a one-way analysis of variance. Different lowercase letters within the same row denote significant differences among dietary treatments according to Tukey’s HSD multiple range test (*p* < 0.05). Absence of letters indicates no significant differences.

**Table 2:** Texture, color, and cooking weight loss in raw and cooked filets of gilthead sea bream (800 g) fed experimental diets.

Parameter	ANOVA (*p*-Value)	FM	PAP	ALT
Raw filets				
Texture				
Hardness (g)	0.746	4293 ± 2115	4618 ± 2533	4694 ± 2683
Adhesiveness (g s)	0.850	−21.34 ± 7.07	−20.57 ± 6.52	−21.45 ± 8.57
Springiness	0.533	0.90 ± 0.04	0.91 ± 0.04	0.91 ± 0.05
Cohesiveness	0.760	1.94 ± 0.65	1.80 ± 0.88	1.89 ± 0.94
Gumminess	0.231	7226 ± 2368	6495 ± 1733	6637 ± 1800
Chewiness	0.348	6554 ± 2236	5968 ± 1710	6057 ± 1744
Resilience	0.559	0.34 ± 0.07	0.35 ± 0.07	0.33 ± 0.06
Color				
L*	0.470	50.70 ± 2.36	50.04 ± 4.01	50.52 ± 2.27
a*	0.058	−1.05 ± 1.14	−0.73 ± 1.13	−0.55 ± 1.18
b*	0.000	9.89 ± 0.89 ^b^	9.36 ± 0.86 ^a^	9.05 ± 1.25 ^a^
C*	0.000	10.01 ± 0.93 ^b^	9.45 ± 0.91 ^a^	9.17 ± 1.11 ^a^
h	0.177	95.94 ± 6.31	94.26 ± 6.55	93.42 ± 9.22
Cooked filets				
Texture				
Hardness (g)	0.596	531 ± 211	527 ± 214	573 ± 226
Adhesiveness (g s)	0.504	−1.45 ± 1.40	−1.43 ± 1.49	−1.85 ± 2.17
Springiness	0.707	0.88 ± 0.02	0.88 ± 0.03	0.88 ± 0.02
Cohesiveness	0.161	1.19 ± 0.17	1.12 ± 0.17	1.13 ± 0.18
Gumminess	0.590	626 ± 254	580 ± 222	629 ± 226
Chewiness	0.598	554 ± 234	511 ± 201	553 ± 207
Resilience	0.008	0.19 ± 0.03 ^b^	0.17 ± 0.03 ^ab^	0.17 ± 0.02 ^a^
Color				
L*	0.036	77.81 ± 2.98 ^b^	77.67 ± 1.97 ^ab^	76.59 ± 3.23 ^a^
a*	0.501	−1.71 ± 0.67	−1.60 ± 0.64	−1.75 ± 0.76
b*	0.000	13.80 ± 1.97 ^a^	14.63 ± 1.73 ^b^	15.18 ± 1.51 ^b^
C*	0.000	13.93 ± 1.90 ^a^	14.73 ± 1.69 ^b^	15.30 ± 1.48 ^b^
h	0.231	97.44 ± 3.64	96.48 ± 2.75	96.67 ± 3.12
Cooking weight loss (%)	0.068	11.95 ± 1.98	11.92 ± 1.96	13.25 ± 1.99

FM: fish meal-based diet; PAP: processed animal protein-based diet; ALT: microbial biomass and insect protein-based diet. Data are presented as means ± SD (*n* = 20). The ANOVA column indicates the *p*-value derived from a one-way analysis of variance. Different lowercase letters within the same row denote significant differences among dietary treatments according to Tukey’s HSD multiple range test (*p* < 0.05). Absence of letters indicates no significant differences.

**Table 3:** Free amino acid content (mg 100 g^−1^, dry weight) of gilthead sea bream (300 g) fed experimental diets.

Free Amino Acids	ANOVA (*p*-Value)	FM	PAP	ALT
Alanine	0.051	6.09 ± 0.71	7.03 ± 1.00	5.15 ± 0.60
Glycine	0.019	11.78 ± 0.34 ^ab^	13.08 ± 2.49 ^a^	8.59 ± 1.10 ^b^
Proline	0.012	2.37 ± 0.15 ^a^	2.31 ± 0.36 ^a^	1.52 ± 0.33 ^b^
Serine	0.015	0.45 ± 0.04 ^b^	0.90 ± 0.29 ^a^	0.45 ± 0.05 ^b^
Threonine	0.017	0.93 ± 0.03 ^b^	1.07 ± 0.17 ^ab^	1.28 ± 0.12 ^a^
Sweet FAAs	0.020	21.62 ± 0.74 ^ab^	24.38 ± 4.25 ^a^	16.99 ± 1.27 ^b^
Aspartic acid	0.309	0.04 ± 0.01	0.05 ± 0.02	0.04 ± 0.01
Glutamic acid	0.077	0.46 ± 0.05	0.76 ± 0.28	0.42 ± 0.08
Umami FAAs	0.083	0.50 ± 0.05	0.81 ± 0.30	0.46 ± 0.08
Arginine	0.060	trace	trace	trace
Histidine	0.013	0.06 ± 0.01 ^ab^	0.08 ± 0.02 ^a^	0.04 ± 0.01 ^b^
Isoleucine	0.987	0.30 ± 0.02	0.30 ± 0.01	0.30 ± 0.06
Leucine	0.517	0.52 ± 0.06	0.59 ± 0.10	0.50 ± 0.13
Lysine	0.037	0.11 ± 0.02 ^b^	0.29 ± 0.08 ^a^	0.19 ± 0.09 ^ab^
Methionine	0.527	0.05 ± 0.01	0.06 ± 0.02	0.05 ± 0.01
Phenylalanine	0.085	0.68 ± 0.05	0.58 ± 0.04	0.57 ± 0.07
Tyrosine	0.192	0.23 ± 0.06	0.34 ± 0.13	0.22 ± 0.01
Valine	0.005	0.38 ± 0.01 ^b^	0.54 ± 0.06 ^a^	0.40 ± 0.06 ^b^
Bitter FAAs	0.110	2.31 ± 0.17	2.77 ± 0.36	2.26 ± 0.31
Asparagine	0.006	0.19 ± 0.01 ^ab^	0.27 ± 0.06 ^a^	0.13 ± 0.02 ^b^
Cysteine	0.036	1.92 ± 0.09 ^ab^	1.80 ± 0.04 ^b^	1.98 ± 0.06 ^a^
Glutamine	0.040	0.10 ± 0.02 ^b^	0.28 ± 0.08 ^a^	0.18 ± 0.09 ^ab^
4-Hydroxyproline	0.000	0.98 ± 0.12 ^a^	1.17 ± 0.01 ^a^	0.52 ± 0.09 ^b^
Methionine sulfoxide	0.010	0.44 ± 0.04 ^a^	0.26 ± 0.08 ^b^	0.45 ± 0.07 ^a^
Tryptophan	0.130	0.09 ± 0.02	0.13 ± 0.04	0.09 ± 0.01
TFAAs	0.021	28.15 ± 0.33 ^ab^	31.88 ± 5.04 ^a^	23.07 ± 1.73 ^b^

FM: fishmeal-based diet; PAP: processed animal protein-based diet; ALT: microbial biomass and insect protein-based diet; TFAAs: total free amino acids. Sweet FAAs: alanine (Ala), glycine (Gly), proline (Pro), serine (Ser), and threonine (Thr). Umami FAAs: aspartic acid (Asp) and glutamic acid (Glu). Bitter FAAs: arginine (Arg), histidine (His), isoleucine (Ile), leucine (Leu), lysine (Lys), methionine (Met), phenylalanine (Phe), tyrosine (Tyr), and valine (Val). Data are presented as means ± SD (*n* = 4). The ANOVA column indicates the *p*-value derived from a one-way analysis of variance. Different lowercase letters within the same row denote significant differences among dietary treatments according to Tukey’s HSD multiple range test (*p* < 0.05). Absence of letters indicates no significant differences.

**Table 4:** Free amino acid content (mg 100 g^−1^, dry weight) of gilthead sea bream (800 g) fed experimental diets.

**Free Amino Acids**	**ANOVA** **(** ***p-*** **Value)**	**FM**	**PAP**	**ALT**
Alanine	0.024	4.03 ± 0.27 ^b^	5.85 ± 1.56 ^a^	4.25 ± 1.45 ^ab^
Glycine	0.000	5.18 ± 0.22 ^b^	9.93 ± 2.58 ^a^	6.47 ± 2.01 ^b^
Proline	0.053	1.90 ± 0.73	2.36 ± 0.86	1.33 ± 0.59
Serine	0.000	0.14 ± 0.03 ^b^	0.25 ± 0.05 ^a^	0.15 ± 0.06 ^b^
Threonine	0.136	0.92 ± 0.06	1.21 ± 0.36	1.29 ± 0.48
Sweet FAAs	0.005	12.16 ± 0.85 ^b^	19.59 ± 5.18 ^a^	13.49 ± 4.45 ^b^
Aspartic acid	0.020	0.02 ± 0.01 ^a^	0.02 ± 0.01 ^ab^	0.02 ± 0.01 ^b^
Glutamic acid	0.074	0.35 ± 0.03	0.41 ± 0.21	0.21 ± 0.19
Umami FAAs	0.074	0.37 ± 0.03	0.43 ± 0.21	0.22 ± 0.20
Arginine	0.022	trace	trace	trace
Histidine	0.000	0.04 ± 0.01 ^b^	0.06 ± 0.01 ^a^	0.04 ± 0.01 ^b^
Isoleucine	0.000	0.12 ± 0.01 ^b^	0.27 ± 0.07 ^a^	0.25 ± 0.05 ^a^
Leucine	0.001	0.10 ± 0.02 ^b^	0.36 ± 0.18 ^a^	0.36 ± 0.10 ^a^
Lysine	0.046	0.09 ± 0.01 ^b^	0.15 ± 0.06 ^a^	0.12 ± 0.03 ^ab^
Methionine	0.000	0.04 ± 0.01 ^b^	0.06 ± 0.01 ^a^	0.06 ± 0.01 ^a^
Phenylalanine	0.081	0.38 ± 0.03	0.50 ± 0.07	0.41 ± 0.16
Tyrosine	0.000	0.09 ± 0.02 ^b^	0.21 ± 0.07 ^a^	0.19 ± 0.04 ^a^
Valine	0.000	0.11 ± 0.02 ^b^	0.24 ± 0.04 ^a^	0.22 ± 0.07 ^a^
Bitter FAAs	0.000	0.97 ± 0.11 ^b^	1.83 ± 0.37 ^a^	1.65 ± 0.30 ^a^
Asparagine	0.000	0.21 ± 0.01 ^b^	0.50 ± 0.15 ^a^	0.26 ± 0.06 ^b^
Cysteine	0.000	1.76 ± 0.07 ^b^	2.02 ± 0.11 ^a^	2.04 ± 0.13 ^a^
Glutamine	0.058	0.09 ± 0.02	0.14 ± 0.05	0.12 ± 0.03
4-Hydroxyproline	0.000	1.32 ± 0.07 ^b^	3.46 ± 1.14 ^a^	1.57 ± 0.56 ^b^
Methionine sulfoxide	0.001	0.11 ± 0.01 ^b^	0.15 ± 0.07 ^b^	0.25 ± 0.07 ^a^
Tryptophan	0.001	0.04 ± 0.01 ^b^	0.09 ± 0.03 ^a^	0.08 ± 0.02 ^a^
TFAAs	0.000	17.03 ± 0.83 ^b^	28.22 ± 5.81 ^a^	19.67 ± 5.27 ^b^

FM: fish meal-based diet; PAP: processed animal protein-based diet; ALT: microbial biomass and insect protein-based diet; TFAAs: total free amino acids. Sweet FAAs: alanine (Ala), glycine (Gly), proline (Pro), serine (Ser), and threonine (Thr). Umami FAAs: aspartic acid (Asp) and glutamic acid (Glu). Bitter FAAs: arginine (Arg), histidine (His), isoleucine (Ile), leucine (Leu), lysine (Lys), methionine (Met), phenylalanine (Phe), tyrosine (Tyr), and valine (Val). Data are presented as means ± SD (*n* = 4). The ANOVA column indicates the *p*-value derived from a one-way analysis of variance. Different lowercase letters within the same row denote significant differences among dietary treatments according to Tukey’s HSD multiple range test (*p* < 0.05). Absence of letters indicates no significant differences.

**Table 5:** Volatile content (% relative abundance) of gilthead sea bream (800 g) fed experimental diets.

Compound	Rt	RI Exp	RI lit	ANOVA	FM	PAP	ALT	Family
1,2-Propanediamine	1.947	600	*	0.002	0.32 ^ab^ ± 0.104	0.52 ^b^ ± 0.041	0.13 ^a^ ± 0.038	Diamine
Acetic acid	6.417	665	663	0.007	4.43 ^b^ ± 0.019	2.17 ^a^ ± 0.002	1.56 ^a^ ± 0.001	Acid
1-Methoxy-2-propanone	6.564	775	770	0.000	10.63 ^b^ ± 0.039	0.55 ^a^ ± 0.001	0.72 ^a^ ± 0.002	Eter
Hexanal	6.782	801	802	0.495	0.74 ± 0.010	0.23 ± 0.000	0.22 ± 0.001	Aldehyde
1-Hexanol	7.177	870	869	0.000	0.62 ^b^ ± 0.001	0.19 ^a^ ± 0.000	0.23 ^a^ ± 0.001	Alcohol
Ethylbenzene	7.419	890	878	0.026	0.80 ^a^ ± 0.001	0.94 ^ab^ ± 0.001	1.17 ^b^ ± 0.002	Aromatic hydrocarbon
m-Xylene	7.610	882	880	0.264	0.77 ± 0.002	0.69 ± 0.002	0.88 ± 0.002	Aromatic hydrocarbon
3,5,5-Trimethyl-1-hexene	7.865	898	900	0.003	1.29 ^b^ ± 0.003	0.74 ^a^ ± 0.001	1.27 ^b^ ± 0.002	Alkene
2-Nonene	8.648	905	902	0.000	2.12 ^b^ ± 0.003	1.28 ^a^ ± 0.001	1.13 ^a^ ± 0.001	Alkene
o-Xylene	8.965	905	905	0.007	0.67 ^a^ ± 0.001	0.87 ^a^ ± 0.001	1.13 ^b^ ± 0.002	Aromatic hydrocarbon
1-Heptanol	9.258	978	972	0.121	0.40 ± 0.002	0.19 ± 0.000	0.28 ± 0.001	Alcohol
*β*-Pinene	9.700	980	978	0.000	7.01 ^b^ ± 0.011	3.67 ^a^ ± 0.003	4.77 ^a^ ± 0.003	Terpene
1-Octen-3-ol	10.177	974	978	0.017	1.36 ^b^ ± 0.004	0.74 ^a^ ± 0.001	1.39 ^b^ ± 0.003	Alcohol
6-Methyl-5-hepten-2-one	10.908	984	985	0.105	0.27 ± 0.001	0.22 ± 0.000	0.19 ± 0.000	Ketone
*β*-Myrcene	11.284	996	998	0.556	4.12 ± 0.009	4.42 ± 0.005	4.63 ± 0.004	Terpene
Octanal	11.569	1002	1000	0.024	0.15 ^a^ ± 0.001	0.32 ^b^ ± 0.001	0.18 ^ab^ ± 0.000	Aldehyde
Hexanoic acid	12.153	1002	1003	0.013	0.16 ^a^ ± 0.000	0.34 ^b^ ± 0.001	0.25 ^ab^ ± 0.000	Acid
Pseudolimonene	12.613	1004	1005	0.001	1.60 ^b^ ± 0.003	0.98 ^a^ ± 0.001	1.49 ^b^ ± 0.001	Terpene
2-Carene	13.068	1005	1006	0.538	0.27 ± 0.001	0.16 ± 0.001	0.37 ± 0.004	Terpene
1,3,5,7-Cyclooctatetraene	13.510	1020	*	0.000	1.50 ^a^ ± 0.003	2.82 ^b^ ± 0.004	2.97 ^b^ ± 0.005	Cyclic hydrocarbon
*t*-*β*-Ocimene	14.304	1020	1023	0.092	4.46 ± 0.011	3.52 ± 0.005	4.68 ± 0.007	Terpene
D-Limonene	14.873	1030	1030	0.004	16.33 ^a^ ± 0.128	18.37 ^a^ ± 0.105	19.57 ^b^ ± 0.048	Terpene
2-Ethyl-1-hexanol	15.245	1038	1034	0.547	1.57 ± 0.003	2.04 ± 0.003	1.62 ± 0.002	Alcohol
Benzyl alcohol	15.627	1040	1034	0.000	0.41 ^a^ ± 0.001	0.90 ^b^ ± 0.001	0.78 ^b^ ± 0.001	Alcohol
Eucalyptol	16.577	1044	1035	0.617	1.06 ± 0.009	2.40 ± 0.018	1.17 ± 0.011	Terpene
*β*-Cymene	17.909	1048	1040	0.000	4.81 ^b^ ± 0.008	2.69 ^a^ ± 0.002	2.89 ^a^ ± 0.002	Aromatic hydrocarbon
1,3-Dichloro-benzene	18.418	1050	1047	0.000	0.44 ^a^ ± 0.001	0.78 ^b^ ± 0.001	0.81 ^b^ ± 0.001	Chlorobenzene
3-Methyl-1,5-pentanediol	18.622	1060	*	0.693	2.80 ± 0.015	4.13 ± 0.009	3.31 ± 0.008	Alcohol
3-(2-Methylpropyl)-cyclohexene	20.337	1062	*	0.202	1.09 ± 0.012	0.16 ± 0.000	0.13 ± 0.000	Cycloalkene
*α*-Terpinolene	20.626	1065	1063	0.537	0.72 ± 0.002	0.67 ± 0.001	0.65 ± 0.001	Terpene
*γ*-Terpinene	20.785	1062	1064	0.379	12.77 ± 0.024	17.57 ± 0.019	15.16 ± 0.023	Terpene
Acetophenone	20.991	1064	1068	0.032	0.40 ^a^ ± 0.002	0.88 ^b^ ± 0.001	0.63 ^ab^ ± 0.001	Ketone
Nonanal	21.986	1100	1102	0.719	2.58 ± 0.014	2.23 ± 0.002	2.32 ± 0.004	Aldehyde
Linalool	22.247	1106	1104	0.381	0.06 ± 0.000	0.06 ± 0.000	0.12 ± 0.001	Terpene
Octanoic acid	22.846	1186	1182	0.000	0.12 ^a^ ± 0.000	0.42 ^b^ ± 0.001	0.23 ^a^ ± 0.000	Acid
Cyclododecane	23.587	1210	1205	0.000	2.40 ^a^ ± 0.010	7.14 ^b^ ± 0.010	7.89 ^b^ ± 0.011	Cycloalkane
Decanal	24.579	1226	1228	0.291	0.27 ± 0.000	0.35 ± 0.001	0.24 ± 0.001	Aldehyde
Nonanoic acid	24.913	1280	1278	0.002	0.16 ^a^ ± 0.001	0.47 ^b^ ± 0.001	0.35 ^b^ ± 0.001	Acid
Tridecane	27.157	1302	1300	0.214	0.14 ± 0.000	0.24 ± 0.001	0.20 ± 0.001	Alkane
Farnesane	27.707	1304	1300	0.110	0.09 ± 0.000	0.16 ± 0.000	0.12 ± 0.000	Terpene
N,N-Dibutyl-formamide	28.038	1305	1310	0.105	0.26 ± 0.002	0.50 ± 0.001	0.46 ± 0.001	Amide
2,7-Dimethyl-octane	28.901	1374	1370	0.380	0.18 ± 0.001	0.14 ± 0.000	0.17 ± 0.000	Alkane
Decanoic acid	29.875	1375	1373	0.050	0.17 ^a^ ± 0.001	0.61 ^b^ ± 0.003	0.32 ^ab^ ± 0.001	Acid
Ethyl decanoate	30.546	1380	1381	0.007	0.10 ^a^ ± 0.000	0.21 ^ab^ ± 0.001	0.27 ^b^ ± 0.001	Ester
Tetradecane	30.722	1402	1400	0.023	0.52 ^a^ ± 0.001	0.88 ^ab^ ± 0.001	0.91 ^b^ ± 0.002	Alkane
Dodecanal	32.263	1405	1402	0.215	0.31 ± 0.001	0.43 ± 0.001	0.51 ± 0.001	Aldehyde
Pentadecane	32.908	1504	1500	0.771	1.79 ± 0.005	2.09 ± 0.004	2.14 ± 0.005	Alkane
2-Undecen-1-ol	34.075	1502	1505	0.141	1.06 ± 0.003	1.44 ± 0.002	1.60 ± 0.003	Alcohol
Methyl dodecanoate	35.415	1528	1525	0.003	0.14 ^a^ ± 0.001	0.34 ^b^ ± 0.001	0.35 ^b^ ± 0.001	Ester
Caryophyllene oxide	36.219	1581	1580	0.000	0.38 ^a^ ± 0.001	1.22 ^b^ ± 0.002	1.12 ^b^ ± 0.002	Sesquiterpene
Hexadecane	39.857	1602	1600	0.008	0.45 ^b^ ± 0.002	0.13 ^a^ ± 0.000	0.26 ^ab^ ± 0.001	Alkane
Heptadecane	42.630	1708	1700	0.067	0.68 ± 0.002	1.11 ± 0.002	1.00 ± 0.001	Alkane
Pristane	45.990	1695	1703	0.000	1.38 ^a^ ± 0.003	3.15 ^b^ ± 0.004	2.67 ^b^ ± 0.003	Alkane
6-Phenylhexanoic acid	49.199	1928	1925	0.025	0.03 ^a^ ± 0.000	0.07 ^b^ ± 0.000	0.04 ^ab^ ± 0.000	Acid
2-Acetylfluorene	51.243	2155	2155	0.000	0.66 ^b^ ± 0.001	0.49 ^a^ ± 0.001	0.34 ^a^ ± 0.001	Ketone
Chemical family								
Acid				0.021	5.06 ^a^ ± 0.019	4.07 ^b^ ± 0.004	2.75 ^c^ ± 0.002	
Alkane				0.015	5.14 ^b^ ± 0.006	7.74 ^a^ ± 0.006	7.35 ^a^ ± 0.007	
Alcohol				0.018	8.23 ^b^ ± 0.016	9.63 ^a^ ± 0.010	9.21 ^a^ ± 0.009	
Aldehyde				0.058	4.04 ± 0.017	3.57 ± 0.002	3.47 ± 0.004	
Alkene				0.020	3.41 ^a^ ± 0.004	2.02 ^b^ ± 0.001	2.40 ^b^ ± 0.002	
Aromatic hydrocarbon				0.013	7.06 ^a^ ± 0.008	5.20 ^c^ ± 0.003	6.07 ^b^ ± 0.004	
Ketone				0.084	1.32 ± 0.002	1.58 ± 0.001	1.16 ± 0.001	
Others				0.015	16.87 ^a^ ± 0.112	13.01 ^b^ ± 0.042	13.74 ^b^ ± 0.040	
Terpene				0.035	48.86 ^b^ ± 0.131	53.18 ^a^ ± 0.108	53.84 ^a^ ± 0.055	

FM: fishmeal-based diet; PAP: processed animal protein-based diet; ALT: microbial biomass and insect protein-based diet. Rt. = retention time (min). RI exp. = experimental lineal retention index. RI lit. = literature (NIST database) lineal retention index. *: tentatively identify. The ANOVA column indicates the *p*-value derived from a one-way analysis of variance. Different lowercase letters within the same row denote significant differences among dietary treatments according to Tukey’s HSD multiple range test (*p* < 0.05). Absence of letters indicates no significant differences.

**Table 6:** Descriptive sensory analysis of cooked gilthead sea bream (300 g) fed experimental diets.

Attributes	ANOVA (*p*-Value)	FM	PAP	ALT
Appearance				
Color	0.139	3.1 ± 1.2	2.4 ± 0.7	2.8 ± 0.9
Color uniformity	0.541	7.4 ± 0.7	7.4 ± 1.2	7.8 ± 1.1
Fat droplets	0.000	8.1 ± 1.9 ^a^	4.6 ± 0.8 ^b^	7.3 ± 1.3 ^a^
Lamellar structure	0.835	3.6 ± 1.7	3.6 ± 1.8	3.2 ± 1.7
Flavor				
Fishy-ID	0.871	8.0 ± 1.9	8.2 ± 1.5	8.3 ± 1.2
Marine	0.230	7.7 ± 1.8	7.0 ± 1.7	8.0 ± 1.3
Fresh	0.488	8.2 ± 0.7	7.8 ± 0.7	8.0 ± 1.1
Fatty	0.589	4.0 ± 1.3	4.1 ± 1.4	4.6 ± 2.0
Earthy	0.160	1.2 ± 0.7	0.9 ± 0.6	0.7 ± 0.6
Vegetal	0.612	0.0 ± 0.1	0.0 ± 0.0	0.0 ± 0.1
Cooked	0.375	2.5 ± 0.6	2.6 ± 0.9	2.3 ± 0.6
Sweetness	0.443	1.8 ± 0.5	2.1 ± 0.7	2.2 ± 1.1
Sourness	0.219	0.1 ± 0.2	0.2 ± 0.4	0.0 ± 0.0
Bitterness	0.179	0.4 ± 0.4	0.8 ± 0.7	0.5 ± 0.5
Saltiness	0.463	2.0 ± 1.2	1.6 ± 1.2	1.4 ± 1.0
Umami	0.000	2.0 ± 0.3 ^b^	2.6 ± 0.5 ^a^	3.0 ± 0.7 ^a^
Metallic	0.520	2.0 ± 1.9	2.5 ± 2.2	1.7 ± 1.6
Rancid/ammonia	0.923	n.d.	n.d.	n.d.
Texture				
Hardness	0.420	5.3 ± 1.4	6.3 ± 1.1	6.8 ± 1.1
Chewiness	0.017	4.6 ± 1.0 ^b^	5.1 ± 1.6 ^ab^	6.2 ± 1.4 ^a^
Adhesiveness	0.754	1.8 ± 1.2	1.5 ± 0.8	1.5 ± 1.0
Juiciness	0.718	7.0 ± 1.1	6.9 ± 0.9	6.7 ± 1.3
Oiliness	0.429	6.8 ± 0.6	6.4 ± 1.6	7.1 ± 1.7
Aftertaste	0.007	6.8 ± 0.4 ^b^	7.4 ± 1.0 ^ab^	7.9 ± 0.9 ^a^

FM: fishmeal-based diet; PAP: processed animal protein-based diet; ALT: microbial biomass and insect protein-based diet; n.d.: non-detected. Data are presented as means ± SD (*n* = 10). The ANOVA column indicates the *p*-value derived from a one-way analysis of variance. Different lowercase letters within the same row denote significant differences among dietary treatments according to Tukey’s HSD multiple range test (*p* < 0.05). Absence of letters indicates no significant differences.

**Table 7:** Descriptive sensory analysis of cooked gilthead sea bream (800 g) fed experimental diets.

Attributes	ANOVA (*p*-Value)	FM	PAP	ALT
Visual				
Color	0.000	2.3 ± 0.2 ^b^	3.6 ± 0.4 ^a^	2.1 ± 0.8 ^b^
Color uniformity	0.936	8.1 ± 0.3	8.2 ± 0.8	8.3 ± 0.8
Fat droplets	0.953	8.1 ± 0.8	7.9 ± 1.6	8.1 ± 1.0
Lamellar structure	0.298	2.6 ± 0.5	2.9 ± 0.2	3.1 ± 0.9
Flavor				
Fish ID	0.283	8.3 ± 0.5	8.0 ± 0.5	7.8 ± 0.6
Marine	0.624	8.1 ± 0.2	7.9 ± 0.8	7.9 ± 0.6
Fresh	0.387	8.1 ± 0.3	7.3 ± 1.4	7.6 ± 1.2
Fatty	0.143	2.9 ± 0.6	3.5 ± 1.6	2.1 ± 1.0
Earthy	0.349	2.2 ± 0.8	1.4 ± 0.6	1.9 ± 1.4
Vegetal	0.202	0.4 ± 0.6	n.d.	0.1 ± 0.2
Cooked	0.804	2.2 ± 0.2	2.4 ± 1.8	1.9 ± 1.3
Sweetness	0.431	1.5 ± 0.7	2.1 ± 0.9	1.8 ± 0.6
Sourness	0.128	0.6 ± 0.4	0.4 ± 0.2	0.2 ± 0.4
Bitterness	0.482	0.1 ± 0.2	0.4 ± 0.7	0.2 ± 0.5
Saltiness	0.487	1.1 ± 0.6	1.4 ± 0.5	1.4 ± 0.7
Umami	0.789	2.6 ± 0.3	2.9 ± 1.2	2.9 ± 1.0
Metallic	0.294	1.0 ± 0.4	1.5 ± 0.7	1.0 ± 0.7
Rancid/ammonia	0.848	0.1 ± 0.2	0.1 ± 0.3	0.1 ± 0.2
Texture				
Hardness	0.046	3.9 ± 1.2 ^b^	5.5 ± 1.8 ^ab^	6.4 ± 1.8 ^a^
Chewiness	0.552	6.7 ± 1.2	6.0 ± 1.9	6.9 ± 1.2
Adhesiveness	0.187	1.1 ± 0.3	1.1 ± 0.6	1.7 ± 0.8
Juiciness	0.242	7.3 ± 0.8	7.8 ± 0.8	6.6 ± 1.8
Oiliness	0.187	7.1 ± 0.6	7.6 ± 0.8	6.4 ± 1.6
Aftertaste	0.979	7.1 ± 0.7	7.1 ± 0.4	7.1 ± 0.8

FM: fish meal-based diet; PAP: processed animal protein-based diet; ALT: microbial biomass and insect protein-based diet; n.d.: non-detected. Data are presented as means ± SD (*n* = 10). The ANOVA column indicates the *p*-value derived from a one-way analysis of variance. Different lowercase letters within the same row denote significant differences among dietary treatments according to Tukey’s HSD multiple range test (*p* < 0.05). Absence of letters indicates no significant differences.

## Data Availability

The original contributions presented in this study are included in the article/Supplementary Materials. Further inquiries can be directed to the corresponding author.

## Supplementary Materials

The following supporting information can be downloaded at: https://www.mdpi.com/article/10.3390/foods15203590/s1, Table S1: Ingredients of experimental diets with a pellet size of 2, 3, 4.5, and 6 mm. Values of pellet size of 2, 3, 4.5, and 6 mm are in bold, red, and regular font, respectively, if variations are present. Otherwise, a unique value is shown; Table S2: Standard amino acid references and calibration curves; Table S3: Chromatographic method for amino acids; Table S4: Amino acid analysis parameters; Table S5: Hedonic evaluation of cooked gilthead sea bream (800 g) fed experimental diets. Figure S1: Filet section selected for physical, chemical and sensory analysis.

## References

[1] Costello C., Cao L., Gelcich S., Cisneros-Mata M.Á., Free C.M., Froehlich H.E., Golden C.D., Ishimura G., Maier J., Macadam-Somer I. (2020). The future of food from the sea. Nature.

[2] Calanche J.B., Beltrán J.A., Arias A.J.H. (2020). Aquaculture and sensometrics: The need to evaluate sensory attributes and the consumers’ preferences. Rev. Aquac..

[3] Casanova-Martínez I., Hernández-López E., Signes-Pastor A.J., Sendra E., Carbonell-Barrachina Á.A., Cano-Lamadrid M. (2025). Abiotic pollutant concentrations in fish: A comparative review of wild-caught and aquaculture sources. Food Control.

[4] Calanche-Morales J.B., Tomás-Vidal A., Cusiyunca Phoco E.R., Martínez-Llorens S., Marquina P.L., Jover-Cerdá M., Roncalés P., Beltrán J.A. (2021). An approach to the Spanish consumer’s perception of the sensory quality of environmentally friendly seabass. Foods.

[5] FAO (2024). The State of World Fisheries and Aquaculture 2024—Blue Transformation in Action.

[6] FAO (2024). Seabass and Seabream: Trade and Prices Are Stable; Supplies Anticipated to Increase (GLOBEFISH Quarterly Species Analysis, June 2024).

[7] Alasalvar C., Taylor K., Zubcov E., Shahidi F., Alexis M. (2002). Differentiation of cultured and wild sea bass (*Dicentrarchus labrax*): Total lipid content, fatty acid and trace mineral composition. Food Chem..

[8] Yildiz M., Şener E., Timur M. (2008). Effects of differences in diet and seasonal changes on the fatty acid composition in fillets from farmed and wild sea bream (*Sparus aurata* L.) and sea bass (*Dicentrarchus labrax* L.). Int. J. Food Sci. Technol..

[9] Marhuenda-Egea F.C., Sanchez-Jerez P. (2025). Metabolomic insights into wild and farmed gilthead seabream (*Sparus aurata*): Lipid composition, freshness indicators, and environmental adaptations. Molecules.

[10] Lupatsch I., Kissil G.W. (2004). Successful replacement of fishmeal by plant proteins in diets for the gilthead seabream, *Sparus aurata* L.. Isr. J. Aquac.-Bamidgeh.

[11] Benedito-Palos L., Saera-Vila A., Calduch-Giner J.A., Kaushik S., Pérez-Sánchez J. (2007). Combined replacement of fish meal and oil in practical diets for fast growing juveniles of gilthead sea bream (*Sparus aurata* L.): Networking of systemic and local components of GH/IGF axis. Aquaculture.

[12] Simó-Mirabet P., Felip A., Estensoro I., Martos-Sitcha J.A., de las Heras V., Calduch-Giner J.A., Puyalto M., Karalazos V., Sitjà-Bobadilla A., Pérez-Sánchez J. (2018). Impact of low fish meal and fish oil diets on the performance, sex steroid profile and male-female sex reversal of gilthead sea bream (*Sparus aurata*) over a three-year production cycle. Aquaculture.

[13] Santigosa E., Brambilla F., Milanese L. (2021). Microalgae oil as an effective alternative source of EPA and DHA for gilthead seabream (*Sparus aurata*) Aquaculture. Animals.

[14] Dawood M.A.O., Koshio S., Esteban M.A. (2018). Beneficial roles of feed additives as immunostimulants in aquaculture: A review. Rev. Aquac..

[15] Naya-Català F., Torrecillas S., Piazzon M.C., Sarih S., Calduch-Giner J., Fontanillas R., Hostins B., Sitjà-Bobadilla A., Acosta F., Pérez-Sánchez J. (2024). Can the genetic background modulate the effects of feed additives? Answers from gut microbiome and transcriptome interactions in farmed gilthead sea bream (*Sparus aurata*) fed with a mix of phytogenics, organic acids or probiotics. Aquaculture.

[16] Álvarez A., Fontanillas R., Hernández-Contreras A., Hernández M. (2020). Partial replacement of fish oil with vegetal oils in commercial diets: The effect on the quality of gilthead seabream (*Sparus aurata*). Anim. Feed Sci. Technol..

[17] Valenzuela A., Sanhueza J., Valenzuela R. (2015). Las microalgas: Una fuente renovable para la obtención de ácidos grasos omega-3 de cadena larga para la nutrición humana y animal. Rev. Chil. Nutr..

[18] Karapanagiotidis I.T., Psofakis P., Mente E., Malandrakis E., Golomazou E. (2018). Effect of fishmeal replacement by poultry by-product meal on growth performance, proximate composition, digestive enzyme activity, haematological parameters and gene expression of gilthead seabream (*Sparus aurata*). Aquac. Nutr..

[19] Maulu S., Langi S., Hasimuna O.J., Missinhoun D., Munganga B.P., Hampuwo B.M., Gabriel N.N., Elsabagh M., Van Doan H., Kari Z.A. (2022). Recent advances in the utilization of insects as an ingredient in aquafeeds: A review. Anim. Nutr..

[20] Piazzon M., Naya-Català F., Pereira G., Estensoro I., Del Pozo R., Calduch-Giner J., Nuez-Ortín W., Palenzuela O., Sitjà-Bobadilla A., Dias J. (2022). A novel fish meal-free diet formulation supports proper growth and does not impair intestinal parasite susceptibility in gilthead sea bream (*Sparus aurata*) with a reshape of gut microbiota and tissue-specific gene expression patterns. Aquaculture.

[21] Aragão C., Gonçalves A.T., Costas B., Azeredo R., Xavier M.J., Engrola S. (2022). Alternative proteins for fish diets: Implications beyond growth. Animals.

[22] Pulido-Rodríguez L.F., Petochi T., Secci G., Lira de Medeiros A.C., Donadelli V., Di Marco P., Di Giacinto F., Marino G., Longobardi A., Capoccioni F. (2026). Commercial-scale evaluation of finishing diet containing poultry by-product and insect meals for *Sparus aurata*: From fish welfare to consumer acceptance. Sustainability.

[23] Holhorea P.G., Moroni F., Estensoro I., Belenguer A., Domingo-Bretón R., Naya-Català F., Calduch-Giner J., Pérez-Sánchez J. (2026). Physiologically mediated responses in gilthead sea bream (*Sparus aurata*) fed sustainable diets: Seasonal growth under warming conditions. Front. Physiol..

[24] González-Hernández M., Portolés T., Mokh S., Sancho J.V., Calduch-Giner J., Nacher-Mestre J., Pérez-Sánchez J., Ibáñez M. (2025). Comprehensive screening of the contaminants in feeds and gilthead sea bream (*Sparus aurata*) fillets by GC and LC coupled to HRMS technique. Microchem. J..

[25] Sendra E., Casanova-Martínez I., Rodríguez-Estrada M., Calduch-Giner J.À., Pérez-Sánchez J., Cano-Lamadrid M. (2026). Evaluating sustainable feed alternatives in *Sparus aurata*: How alternative proteins and oils maintain EPA+DHA content and improve human health lipid indices. Foods.

[26] Rodríguez-Soriano M., Solivella-Poveda A.M., Ropero A.B., Beltrá M., Cano-Lamadrid M., Sendra E. (2026). Metabolic and microbial stability of protein-fortified fermented dairy drinks: Effect of base and storage. Appl. Food Res..

[27] Wang X., Dong Y., Huang Y., Tian H., Zhao H., Wang J., Zhou J., Liu W., Cao X., Li X. (2024). Docosahexaenoic acid-enriched diet improves the flesh quality of freshwater fish (*Megalobrama amblycephala*): Evaluation based on nutritional value, texture and flavor. Food Chem..

[28] Matos E., Gonçalves A., Bandarra N., Colen R., Nunes M.L., Valente L.M., Dinis M.T., Dias J. (2012). Plant proteins and vegetable oil do not have detrimental effects on post-mortem muscle instrumental texture, sensory properties and nutritional value of gilthead seabream. Aquaculture.

[29] Sarmiento P., Castro P.L., Ginés R. (2025). Impact of alternative feed ingredients and feeding strategies on growth, muscle morphology, and fillet quality of genetically selected gilthead seabream (*Sparus aurata*) in a long-term feeding trial. Animals.

[30] Iaconisi V., Secci G., Sabatino G., Piccolo G., Gasco L., Papini A.M., Parisi G. (2019). Effect of mealworm (*Tenebrio molitor* L.) larvae meal on amino acid composition of gilthead sea bream (*Sparus aurata* L.) and rainbow trout (*Oncorhynchus mykiss* W.) fillets. Aquaculture.

[31] Piccolo G., Iaconisi V., Marono S., Gasco L., Loponte R., Nizza S., Bovera F., Parisi G. (2017). Effect of *Tenebrio molitor* larvae meal on growth performance, in vivo nutrients digestibility, somatic and marketable indexes of gilthead sea bream (*Sparus aurata*). Anim. Feed Sci. Technol..

[32] Damir N., Yıldız M., Ofori-Mensah S., Aydın I. (2025). Effect of poultry by-product meal as replacement for fish meal in diets of gilthead seabream (*Sparus aurata*) juveniles. Turk. J. Fish. Aquat. Sci..

[33] Zhumakadyrova S., Mazhitova A. (2024). The free amino acid profiling of milk- and cereal-based Kyrgyz ethnic fermented beverages and their contribution to taste formation. Food Sci. Appl. Biotechnol..

[34] Podduturi R., Petersen M.A., Mahmud S., Rahman M.M., Jørgensen N.O. (2017). Potential contribution of fish feed and phytoplankton to the content of volatile terpenes in cultured pangasius (*Pangasianodon hypophthalmus*) and tilapia (*Oreochromis niloticus*). J. Agric. Food Chem..

[35] Shahidi F. (2001). Headspace volatile aldehydes as indicators of lipid oxidation in foods. Adv. Exp. Med. Biol..

[36] Grebenteuch S., Kroh L.W., Drusch S., Rohn S. (2021). Formation of secondary and tertiary volatile compounds resulting from the lipid oxidation of rapeseed oil. Foods.

[37] Grigorakis K., Fountoulaki E., Giogios I., Alexis M. (2009). Volatile compounds and organoleptic qualities of gilthead sea bream (*Sparus aurata*) fed commercial diets containing different lipid sources. Aquaculture.

[38] Oliva-Teles A., Enes P., Peres H., Davis D.A. (2015). Replacing fishmeal and fish oil in industrial aquafeeds for carnivorous fish. Feed and Feeding Practice in Aquaculture.

[39] Alexi N., Fountoulaki E., Grigorakis K. (2016). Quality of reared gilthead sea bream (*Sparus aurata*) during ice storage, as affected by dietary fish oil substitution; an instrumental and sensory designation approach. Aquac. Res..

[40] Larsen D., Quek S.Y., Eyres L. (2010). Effect of cooking method on the fatty acid profile of New Zealand king salmon (*Oncorhynchus tshawytscha*). Food Chem..

[41] Panayotova V., Peycheva K., Hristova T., Dobreva D.A., Stoycheva T., Stancheva R., Georgieva S., Andreev E., Nikolova S., Pancheva R. (2026). From raw to cooked: Proximate composition, fatty acids and fat-soluble vitamins in bluefish (*Pomatomus saltatrix*) from the Black Sea. Foods.

[42] Xiang H., Song W., Hao S., Sun-Waterhouse D. (2026). Unlocking the potential of taste and taste-active peptides: Bridging structural characteristics and biological activities with food and pharmaceutical applications. J. Future Foods.

[43] Mu R., Chen J. (2023). Oral bio-interfaces: Properties and functional roles of salivary multilayer in food oral processing. Trends Food Sci. Technol..

[44] Zhao K., Xu Z., Li Y., Quan Q., Xie Y., Wang J., Sun B., Pu D. (2026). Recent advances in understanding the modulatory mechanisms of saliva in flavor perception. Food Chem..

