# Supplementary material for: Evaluating the Sensory Profile and Quality Traits of Sparus aurata Fed Sustainable Diets
Source: Foods. 2026 Oct 10;15(20):3590. doi: 10.3390/foods15203590 (PMC13703060; doi:10.3390/foods15203590)
Supplement: Supplementary file 1 [file foods-15-03590-s001.zip › foods-4572907-supplementary.pdf]

Table S1. Ingredients of experimental diets with pellet sizes of 2, 3, 4.5, and 6 mm. Values for pellet sizes of 2, 3, 4.5, and 6 mm are in bold, red, and regular font, respectively, if variations are present. Otherwise, a unique value is shown.

Ingredients category	Ingredient (%)	Diets		
		FM	PAP	ALT
Marine	Fishmeal Super Prime	**17** - 5 - 5	0	0
	Fishmeal 60	**5** - 10 - 10	**5** - 0 - 0	**5** - 0 - 0
	Fish oil	**5.4** - 6.65 - 6.65	**5.4** - 6.65 - 6.65	**5.4** - 6.65 - 6.65
Vegetal	Corn gluten meal	**7** - 8 - 8	**7** - 8 - 8	**7** - 8 - 8
	Soybean meal 44	**9** - 12 - 12	**9** - 12 - 12	**9** - 12 - 12
	Sunflower meal 40	**4.5** - 7.5 - 7.5	**4.5** - 7.5 - 7.5	**4.5** - 7.5 - 7.5
	Wheat meal	**11.28** - 9.58 - 8.58	**11.73** - 11.58 - 10.88	**8.88** - 8.88 - 8.38
	Whole peas	5	5	5
	Pea starch (raw)	2	2	2
	Rapeseed oil	**6.9** - 7.4 - 7.2	**0** - 5.5 - 5	**6.05** - 7.5 - 7.1
By-products	Poultry meal	12	**25** - 17 - 17	12
	Feathermeal hydrolysate	**5** - 7.25 - 7.25	**5** - 9.75 - 9.5	**5** - 7.25 - 7.25
	Porcine blood meal	5	8	5
	Fish protein hydrolysate	**3** - 0 - 0	**3** - 0 - 0	**3** - 0 - 0
	Salmon oil	0	**7.2** - 3 - 3	0
Novel alternative ingredients	Insect meal (PROTE - IN HP55)	0	0	**12** – 8.5 - 8
	Microbial biomass (Aminopro NT70)	0	0	**6** - 5 - 5
	DHA Algae oil (Veramaris)	0	0	**0.9** - 0.4 - 0.4
Nutrients supplementation[1]	Vitamin and mineral premix[2]	1	1	1
	Vitamin C35	**0** - 0 - 0.05	**0** - 0 - 0.05	**0** - 0 - 0.05
	Vitamin E50	**0.1** - 0.1 - 0.15	**0.1** - 0.1 - 0.15	**0.1** - 0.1 - 0.15
	Antioxidant	0.2	0.2	0.2
	Sodium propionate	0.1	0.1	0.1
	MAP (Monoamonium phosphate)	**0.5** - 0.8 - 0.8	**0.75** - 1.9 - 2.15	**1.55** - 2.1 - 2.3
	L - Lysine HCl 99%	**0** - 0.4 - 0.4	**0** - 0.6 - 0.6	**0.3** - 0.7 - 0.7
	DL - Methionine	0	**0** - 0.1 - 0.1	**0** - 0.1 - 0.1
	Nucleotides (Nucleoforce AQUA)	**0** - 0 - 0.2	**0** - 0 - 0.2	**0** - 0 - 0.2
	L - Taurine	**0** - 0 - 0.4	**0** - 0 - 0.4	**0** - 0 - 0.4
	Yttrium oxide	0.02	0.02	0.02
	Rapeseed lecithin liquid	**0** - 0 - 0.5	**0** - 0 - 0.5	**0** - 0 - 0.5

[1]Presence of contaminants in this ingredient category was not analyzed; [2]Vitamin and mineral premix: Vitamins (IU or mg/kg diet): DL - alpha tocopherol acetate, 565 mg; sodium menadione bisulphate, 23.5 mg; retinyl acetate, 21,750 IU; DL - cholecalciferol, 4,640 IU; thiamine, 28.3 mg; riboflavin, 28.6 mg; pyridoxine, 22 mg; cyanocobalamin, 0.1 mg; nicotinic acid, 205 mg; folic acid, 14 mg; ascorbic acid, 935 mg; inositol, 465 mg; biotin, 2.9 mg; calcium pantothenate, 95.5 mg; choline chloride, 1070 mg; betaine, 465 mg. Mineral (g or mg/kg diet): Na, 3 g; Mg, 1.4 g; K, 5.9 g; Cu, 15.6 mg; Fe, 240 mg; I, 0.95 mg; Mn, 19.9 mg; Se, 0.5 mg; Zn, 48 mg.

Table S2. Standard amino acid references and calibration curves.

Amino acid	CAS	Supplier	References	Equation	R^2
Asparagine	70-47-3	Pharmaceutical Secondary Standard; Certified Reference Material, Sigma-Aldrich, GmbH, Switzerland	51363	y = 797956x + 84545	0.99
Aspartic acid	56-84-8	Pharmaceutical Secondary Standard; Certified Reference Material, Sigma-Aldrich, GmbH, Switzerland	PHR1104	y = 1E+06x + 176355	0.985
Serine	56-45-1	TargetMOl, Linz, Austria (Purity: 99.20%-99.31%)	TM-T202790	y = 2E+06x + 256156	0.9825
Alanine	56-41-7	TargetMOl, Linz, Austria (Purity: 99.83%-99.96%)	TM-TJO2729	y = 2E+06x + 166145	0.9911
Glycine	56-40-6	Alfa Aesar, Kandel, Germany (Purity:99%)	02-A13816	y = 384708x + 39664	0.9893
Glutamine	56-85-9	USP, Madrid, Spain (Reference Standard, Purity 99%)	292419	y = 1E+07x + 371000	0.9966
Threonine	72-19-5	TargetMOl, Linz, Austria (Purity: 98.87%-99.93%)	TM-T202777	y = 2E+06x + 148222	0.9899
Cysteine	52-90-4	TargetMOl, Linz, Austria (Purity: 99.59%-99.89%)	TM-T202721	y = 40473x - 7778,1	0.9281
Methionine sulfoxide	62697-73-8	TargetMOl, Linz, Austria (Purity:99,98%)	TM-TI9418	y = 795063x + 144803	0.926
Glutamic acid	56-86-0	TargetMOl, Linz, Austria (Purity: 99.88%-99.94%)	TM-T2A2497	y = 4E+06x + 304197	0.9924
Proline	147-85-3	Pharmaceutical Secondary Standard; Certified Reference Material, Sigma-Aldrich, GmbH, Switzerland	PHR1332	y = 7E+06x + 614565	0.9948
Lysine	56-87-1	TargetMOl, Linz, Austria (Purity: 99.88%-99.94%)	TM-T2A2498	y = 1E+07x + 338871	0.997
Histidine	71-00-1	TargetMOl, Linz, Austria (Purity: 99.62%-99.92%)	TM-T2A2532	y = 6E+06x + 186115	0.9952
Arginine	200-811-1	TargetMOl, Linz, Austria (Purity: 99.70%-99.98%)	TM-T3S0364	y = 3E+07x - 14335	0.9994
Valine	72-18-4	Pharmaceutical Secondary Standard; Certified Reference Material, Sigma-Aldrich, GmbH, Switzerland	PHR1172	y =9E+06x + 283292	0.9944
Methionine	63-68-3	TargetMOl, Linz, Austria (Purity: 99.84%-99.91%)	TM-T4844	y =1E+07x - 92626	0.9979
Tyrosine	60-18-4	TargetMOl, Linz, Austria (Purity: 99.63%-99.82%)	TM-T0493	y = 7E+06x + 429860	0.9964
Leucine	328-39-2	Pharmaceutical Secondary Standard; Certified Reference Material, Sigma-Aldrich, GmbH, Switzerland	PRH1105	y =9E+06x + 462786	0.9965
Phenylalanine	63-91-2	TargetMOl, Linz, Austria (Purity: 99.37%-99.91%)	TM-T3377	y = 2E+07x + 1E+06	0.9957
Tryptophan	73-22-3	Certified reference material, TraceCERT, Sigma-Aldrich, GmbH, Switzerland	51145	y = 7E+06x + 188163	0.9989
Isoleucine	73-32-5	Pharmaceutical Secondary Standard; Certified Reference Material, Sigma-Aldrich, GmbH, Switzerland	PHR1099	y = 9E+06x + 427184	0.9973
4-Hydroxyproline	51-35-4	Pharmaceutical Secondary Standard; Certified Reference Material, Sigma-Aldrich, GmbH, Switzerland	PHR1939	y = 3E+06x + 290590	0.9889

Table S3. Chromatographic method for amino acids.

Analytical conditions			
Analytical Column	Discovery HS F5-3 (L × I.D. 15 cm × 2.1 mm, 3 µm), Sigma-Aldrich		
Mobile Phase A	0.1% Formic acid-Water		
Mobile Phase B	0.1% Formic acid-Acetonitrile		
Flow Rate	0.25 mL/min		
Injection Volume	3 µL		
Column Oven Temperature	40 ºC		
Gradient Program			
Step	Time (min)	Mobile Phase A (%)	Mobile Phase B (%)
0	0	100	0
1	2	100	0
2	5	75	25
3	11	65	35
4	15	5	95
5	20	5	95
6	20.1	100	0
7	25	100	0
MS Conditions			
Nebulizing Gas Flow Rate	3.0 L/min		
Drying Gas Flow Rate	10.0 L/min		
Heating Gas Flow Rate	10.0 L/min		
Interface Temperature	300 ºC		
DL Temperature	250 ºC		
Block Heater Temperature	400 ºC		
Ionization Mode	ESI		

Table S4. Amino acid analysis parameters

Amino acids	Polarity	Target ion	Confirmation ion	CAS
Asparagine	(+)	133.10>87.15	133.10>28.05	3130-87-8
Aspartic acid	(+)	134.00>74.05	134.00>88.10	56-84-8
Serine	(+)	105.90>60.10		56-45-1
Alanine	(+)	89.90>44.10		56-41-7
Glycine	(+)	75.90>30.15		56-40-6
Glutamine	(+)	147.10>84.15	147.10>130.10	56-85-9
Threonine	(+)	120.10>74.15	120.10>56.05	72-19-5
Cysteine	(+)	122.00>76.05	122.00>59.00	52-90-4
Methionine sulfoxide	(+)	166.00>74.10	166.00>55.95	62697-73-8
Glutamic acid	(+)	147.90>84.10	147.90>56.10	56-86-0
Proline	(+)	116.10>70.15	116.10>28.05	147-85-3
Lysine	(+)	147.10>84.10		56-87-1
Histidine	(+)	155.90>110.10	155.90>56.10	71-00-1
Arginine	(+)	175.10>70.10	175.10>60.10	200-811-1
Valine	(+)	118.10>72.15	118.10>55.05	72-18-4
Methionine	(+)	149.90>56.10	149.90>104.10	59-51-8
Tyrosine	(+)	182.10>136.10	182.10>91.10	60-18-4
Leucine	(+)	132.10>86.05	132.10>30.05	328-39-2
Phenylalanine	(+)	166.10>120.10	166.10>103.10	63-91-2
Tryptophan	(+)	205.10>188.15	205.10>146.10	73-22-3
Isoleucine	(+)	132.10>86.20	132.10>69.15	73-32-5
4-Hydroxyproline	(+)	132.10>86.05	132.10>68.05	51-35-4

Table S5. Hedonic evaluation of cooked gilthead sea bream (800 g) fed experimental diets.

Attributes	**ANOVA**	**FM**	**PAP**	**ALT**
Appearance				
Overall Appearance	0.092	6.0 ± 2.5	6.6 ± 2.4	5.8 ± 2.6
Color	0.674	5.6 ± 2.8	5.9 ± 2.6	5.7 ± 2.6
Surface Oiliness	0.931	5.7 ± 2.7	5.7 ± 2.4	5.8 ± 2.5
Odor				
Fish/fresh/marine	0.394	6.5 ± 2.4	7.0 ± 2.5	6.6 ± 2.1
Oily	0.877	5.7 ± 2.2	5.8 ± 2.2	5.9 ± 2.1
Metallic	0.853	5.1 ± 2.1	5.3 ± 2.1	5.1 ± 2.5
Flavor				
Fish/fresh/marine	0.117	6.5 ± 2.5	7.2 ± 2.3	6.6 ± 2.5
Oily	0.001	5.6 ± 2.3 b	7.0 ± 2.1 a	6.2 ± 2.7 ab
Metallic	0.932	5.4 ± 2.0	5.5 ± 1.8	5.6 ± 2.5
Umami	0.920	6.1 ± 2.0	6.3 ± 1.9	6.2 ± 2.2
Texture				
Chewiness	0.061	5.5 ± 3.0	6.6 ± 2.6	5.8 ± 3.0
Juiciness	0.152	6.1 ± 2.8	6.7 ± 2.7	5.9 ± 2.9
Oiliness	0.001	5.8 ± 2.3 b	7.0 ± 2.1 a	5.8 ± 2.6 b
Aftertaste	0.015	6.0 ± 2.3 b	7.0 ± 2.5 a	6.9 ± 2.5 a

FM: Fish meal-based diet; PAP: processed animal proteins-based diet; ALT: microbial biomass and insect protein-based diet. Data are presented as means ± SD (n = 81). The ANOVA column indicates the p-value derived from a one-way analysis of variance. Different lowercase letters within the same row denote significant differences among dietary treatments according to Tukey's HSD multiple range test ($p < 0.05$). Absence of letters indicates no significant differences.

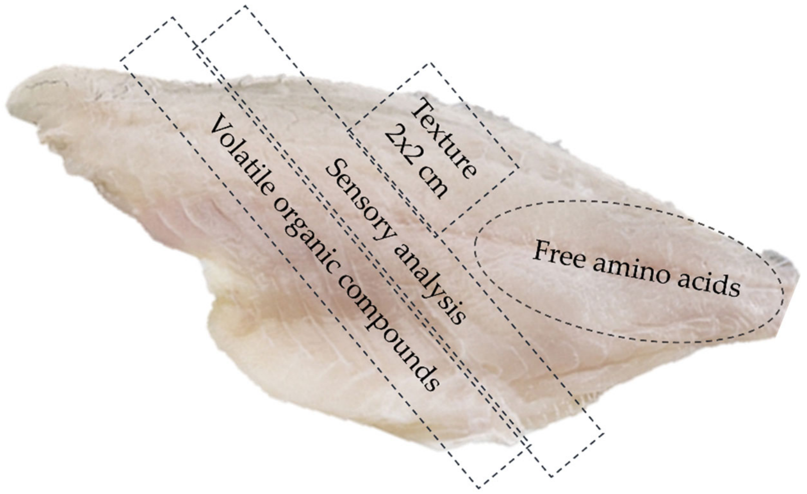

Figure S1. Fillet section selected for physical, chemical, and sensory analysis.
